# Effects of Natural Rhamnolipid Mixture on Dioleoylphosphatidylcholine Model Membrane Depending on Method of Preparation and Sterol Content

**DOI:** 10.3390/membranes13010112

**Published:** 2023-01-15

**Authors:** Konstantin Potapov, Alexander Gordeev, Liliya Biktasheva, Maya Rudakova, Artem Alexandrov

**Affiliations:** 1 Department of Molecular Physics, Institute of Physics, Kazan Federal University, 420011 Kazan, Russia; 2 Institute of Environmental Sciences, Kazan Federal University, 420011 Kazan, Russia; 3 Institute of Information Technology and Intelligent Systems, Kazan Federal University, 420011 Kazan, Russia

**Keywords:** rhamnolipids, model membranes, liposomes, sterols, NMR, ATR-FTIR

## Abstract

Rhamnolipids as biosurfactants have a potentially wide range of applications, for example, as “green” surfactants or components of drug delivery systems, which is associated with the features of their interaction with cell membranes. However, as noted in the literature, those kind of features have not been sufficiently studied now. This paper presents a study of the interaction of a natural mixture of rhamnolipids produced by bacteria of the rhizosphere zone of plants *Pseudomonas aeruginosa* with model membranes—liposomes based on dioleoylphosphatidylcholine (DOPC), depending on the method of their preparation and the content of sterols—ergosterol, cholesterol, lanosterol. Liposomes with rhamnolipids were prepared by two protocols: with film method from a mixture of DOPC and rhamnolipids; with film method from DOPC and injection of water solution of rhamnolipids. Joint analysis of the data of ${}^{31}$P NMR spectroscopy and ATR-FTIR spectroscopy showed that in the presence of rhamnolipids, the mobility of the head group of the DOPC phospholipid increases, the conformational disorder of the hydrophobic tail increases, and the degree of hydration of the C=O and P=O groups of the phospholipid decreases. It can be assumed that, when prepared from a mixture, rhamnolipids are incorporated into the membrane in the form of clusters and are located closer to the middle of the bilayer; while when prepared by injection, rhamnolipid molecules migrate into the membrane in the form of individual molecules and are located closer to the head part of phospholipids. The sterol composition of the model membrane also affects the interaction of rhamnolipids with the membrane. Here it is worth noting the possible presence of type of interaction between rhamnolipids and ergosterol differ from other investigated sterols, due to which rhamnolipid molecules are embedded in the area where ergosterol is located.

## 1. Introduction

Biosurfactants are surface-active substances (surfactants) secreted by various microorganisms: bacteria, yeasts and molds [1,2,3]. These substances are extremely diverse in their structure: glycolipids, lipoamino acids, lipoproteins, biopolymers, phospholipids, fatty acids, etc. [4]. They are distinguished from artificial surfactants by their low toxicity, high biodegradability, and efficiency in a wide range of environmental conditions (temperature, salinity, pH) [1,3,4]. For these reasons, much attention is paid to the study of biosurfactants as a replacement for chemically synthesized surfactants [5].

Rhamnolipids (RL) are one of the representatives of the class of biosurfactants produced by bacteria. The most common producers of this type of biosurfactant are *Pseudomonas aeruginosa, P. chlororaphis, Burkholderia thailandensis* [1,4,6,7,8]. Depending on the production conditions, it is possible to obtain different types of rhamnolipids in structure and properties, of which about 60 are now known [1]. From a practical point of view, rhamnolipids have a number of advantages over other surfactants: high production efficiency and the ability to use various nutrient media for bacteria, including secondary raw materials [1,2,9,10,11]. This primarily determines the interest in rhamnolipids as “green” surfactants that can be used for emulsification in various technological processes, for example, in oil production [1,3,4,5,12]. However, as many studies have shown, rhamnolipids have a wide range of different biological effects. They reduce the growth rate of various microorganisms [13,14,15,16,17,18], activate plant immune responses [16,19,20], demonstrate lytic activity against phytopathogens [21,22], increase sensitivity to various drugs [5,23] and even have antitumor activity [24]. According to modern studies ramnolipids are non-toxic [25] or have low toxicity [26,27]. The combination of these qualities makes it possible to use rhamnolipids as components of drugs and their delivery systems, biocontrol agents in agriculture [19,28].

The described wide range of biological effects caused by RL is associated with the specifics of their interaction with cell membranes, which is confirmed by the observation of changes in the physicochemical characteristics of model membranes in the presence of RL [29,30,31,32], as well as the influence of the membrane composition on the certain effect of the addition of rhamnolipids [33,34]. In this case, a special role in the interaction of RL with the membrane is played by its sterol composition [28,34], since sterols are the second component of the membrane in terms of quantitative representation [35].

To date, a number of sources [12,28,32,33] note that there is insufficient knowledge of the mechanisms of interaction of rhamnolipids with cell membranes. For example, firstly, studies on model membranes did not affect the study of the interaction of rhamnolipids with completely unsaturated phospholipids [28]. Secondly, it is known that rhamnolipids are able to integrate into membranes independently [12], and in the literature, there are two approaches to the experiment: preparation of liposomes from a mixture of lipids to which rhamnolipids are added, and addition of rhamnolipids to an aqueous dispersion of already prepared liposomes, such as this done in a study on the leakage of carboxyfluorescein from liposomes [34]. Studies of the influence of the method of introducing rhamnolipids into samples on the results of the experiment have not yet been carried out. Thirdly, it should be noted that most of the studies were carried out on highly purified samples of rhamnolipids, and natural mixtures of rhamnolipids, the practical application of which is promising, remain without due attention [28].

In present work were studied the effect of a natural mixture of rhamnolipids on the model membrane structure based on the unsaturated phospholipid dioleoylphosphatidylcholine (DOPC), depending on the method of introducing rhamnolipids and the sterol composition of the membrane. The use of DOPC and sterols: ergosterol and cholesterol makes it possible to model the membrane of fungi and higher animals with sufficient quality [36], and the ratio of components in the creation of model membranes in this work is close to the ratios realized in nature [28]. Lanosterol was chosen as the third representative of the sterol series as a metabolic precursor of ergosterol and cholesterol [37].

## 2. Materials and Methods

### 2.1. Materials

**Figure 1:**
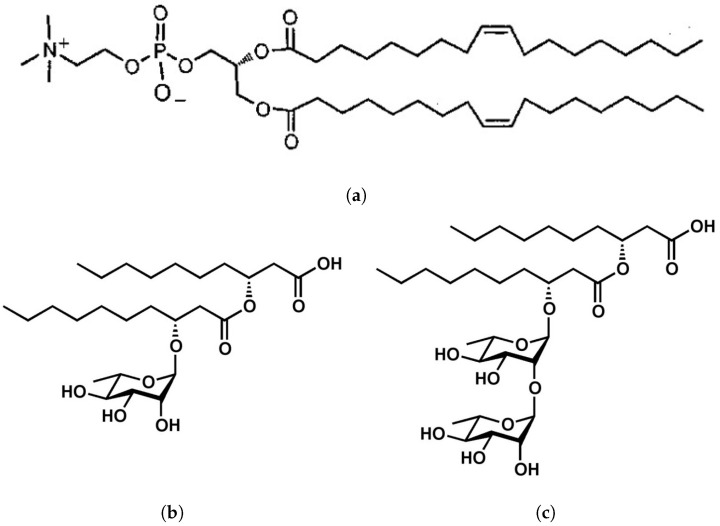
Structural chemical formulas of dioleoylphosphatidylcholine (**a**), mono-rhamnolipid (**b**), di-rhamnolipid (**c**).

Liposomes were prepared from DOPC produced by Avanti Polar Lipids (Alabaster, AL, USA). As rhamnolipids, a natural mixture of mono- and di-rhamnolipids (α-L-rhamn opyranosyl-β-hydroxydecanoyl-β-hydroxy-deconate and 2-O-α-L-rhamnopyranosyl-α-L-rhamnopyranosyl-β-hydroxydecanoyl-β−hydroxydeconate), synthesized by bacteria of the species *Pseudomonas aeruginosa*, manufactured by AGAE Technologies LLC (Corvallis, OR, USA) was used. The purity of the rhamnolipids mixture is 90%, and the ratio of mono- and di-rhamnolipids is 40:60 (average molar mass 591 g/mol). The structural chemical formulas of DOPC and the rhamnolipids used are shown in Figure 1.

Ergosterol, cholesterol, and lanosterol produced by Avanti Polar Lipids were used to modify the sterol composition of liposomes.

### 2.2. Sample Preparation

Four types of samples were studied in this work: liposomes from pure DOPC; liposomes from DOPC with the addition of rhamnolipids; liposomes from DOPC and one of the sterols (ergosterol, lanosterol, cholesterol); liposomes from DOPC, one of the sterols and with the addition of rhamnolipids. Liposomes were obtained using the film preparation method [38,39,40]. The addition of rhamnolipids was carried out by two different protocols: preparation of liposomes from a mixture of DOPC and rhamnolipids; injection of an aqueous solution of rhamnolipids into already prepared liposomes. For both protocols 70 mg of DOPC were used, sterols and rhamnolipids were added in amount to achieve molar ratios of DOPC + sterol/rhamnolipid—25:1 and DOPC/sterol—75:25, which are close to natural ratios (see, for example, [29,37]).

In the first protocol of adding rhamnolipids, a dry mixture of the required composition (DOPC/DOPC + rhamnolipid/DOPC + sterol) was dissolved in a mixture of non-polar solvents: chloroform and methanol (2:1 *v:v*), so that the concentration of lipids in the solution was 10 mg/mL. The solvent was removed from the sample using a rotary evaporator at a pressure of 0.2 bar and a flask rotation speed of 100 rpm until visible solvent residues disappeared (more than 3 h for all samples). To remove traces of solvent, the film flasks were placed in a lyophilizer at −3.9 ${}^{\circ }$C (25 ${}^{\circ }$F) for more than 16 h. After removing the solvent water was added to the finished films so that the concentration of lipids in water was 150 mmol/L, then the flask was vortexed for about half an hour until the lipids were transferred completely from the film on the flask wall into water, where film fragments could close into liposomes.

To homogenize the size of liposomes and reduce their multilamellarity, the freeze-thaw technique was used [28,41], which consisted of four cycles of freezing at a temperature of −80 ${}^{\circ }$C for 15 min and thawing at a temperature of +40 ${}^{\circ }$C for 10 min and one step of additional shaking for a minute. As revealed by freeze-fracture electron microscopy, multilamellar vesicles fragmentation into small unilamellar vesicles takes place progressively: a homogeneous population of unilamellar vesicles requires between five and ten cycles of freeze-thawing. An aqueous dispersion of liposomes was stored in a refrigerator at a temperature of −4 ${}^{\circ }$C for at least a day until all measurements were taken to stabilize the size [38]. The described method of obtaining liposomes gives an aqueous dispersion of multilamellar and unilamellar liposomes with a submicron size [41].

In the second protocol of adding rhamnolipids, DOPC/DOCP + sterol liposomes were prepared according to the first protocol; an aqueous solution of rhamnolipids was injected into already prepared liposomes after the stage of liposome size stabilization; after injection, the samples were also kept at a temperature of −4 ${}^{\circ }$C for at least 12 h.

Thus, the first and second types of samples differ in the method of introducing rhamnolipids, and based on their comparison, it is possible to draw conclusions about the effect of the method of introducing rhamnolipids on the interaction of rhamnolipids with the membrane. The third and fourth types of samples are distinguished by the absence/presence of a sterol component in the membrane; based on a comparison of data for these types of samples, one can judge the effect of the sterol composition of the membrane on the interaction of rhamnolipids with the membrane.

### 2.3. NMR Experiments

${}^{31}$P NMR spectroscopy is one of the most informative methods for studying the structure, dynamics, and phase state of aggregates of phospholipid molecules [42]. In the present work, model DOPC membranes were studied in a natural aqueous environment. This does not allow using ${}^{31}$P MAS NMR and resolving signals from chemically nonequivalent phosphorus groups, however, the shape of the anisotropic spectrum of phosphorus makes it possible to draw conclusions about the phase states (flat bilayer, micelles, vesicles) of phospholipid aggregates [43], as well as their changes with the addition of rhamnolipids.

${}^{31}$P NMR spectra were obtained on a Bruker AVANCE III 400 spectrometer at a resonant frequency of 162.03 MHz by recording Free Induction Decay and subsequent Fourier transform. The duration of the RF-pulse was 6.75 μs, the repetition time was 2.5 s, and the number of accumulations was 256. The spectra were measured at different sample temperatures: 20–40 ${}^{\circ }$C with a step of 5 degrees. The spectra were approximated using the DMfit software, in which the parameters of chemical shift anisotropy (CSA) were determined using the minimization of a quadratic distance between the simulated and experimental spectra with an iterative constrained gradient protocol involving the partial derivatives of all parameters in the line shape model [44,45]. Examples of spectra approximation are given in Supplementary materials (Figure S1).

### 2.4. ATR-FTIR Experiments

In addition to the NMR method, which is sensitive to changes in the local mobility and environment of the head (hydrophilic) part of the phospholipid molecules, FTIR spectroscopy provides information on the vibrational frequencies of the chemical groups of both the hydrophilic and hydrophobic part of the phospholipid molecules; the change in vibration frequencies allows one to draw conclusions about the incorporation of rhamnolipid molecules into the model membrane.

FTIR spectra were obtained in the ATR mode (attenuated total reflection) on a Bruker LUMOS spectrometer at a temperature of 22 ${}^{\circ }$C from 15 μL of the sample. The spectra were recorded in the range from 3100 cm${}^{-1}$ to 900 cm${}^{-1}$ with a spectral resolution of 4 cm${}^{-1}$. Each spectrum is an average of 1024 spectra. Before measuring the experimental spectra, the spectrum was measured from 15 μL of water for subsequent subtraction. The spectra processing, including the determination of the frequency of the absorption peak, was carried out using the OPUS software.

## 3. Results and Discussion

### 3.1. Influence of the Method of Preparation of Liposomes

**Figure 2:**
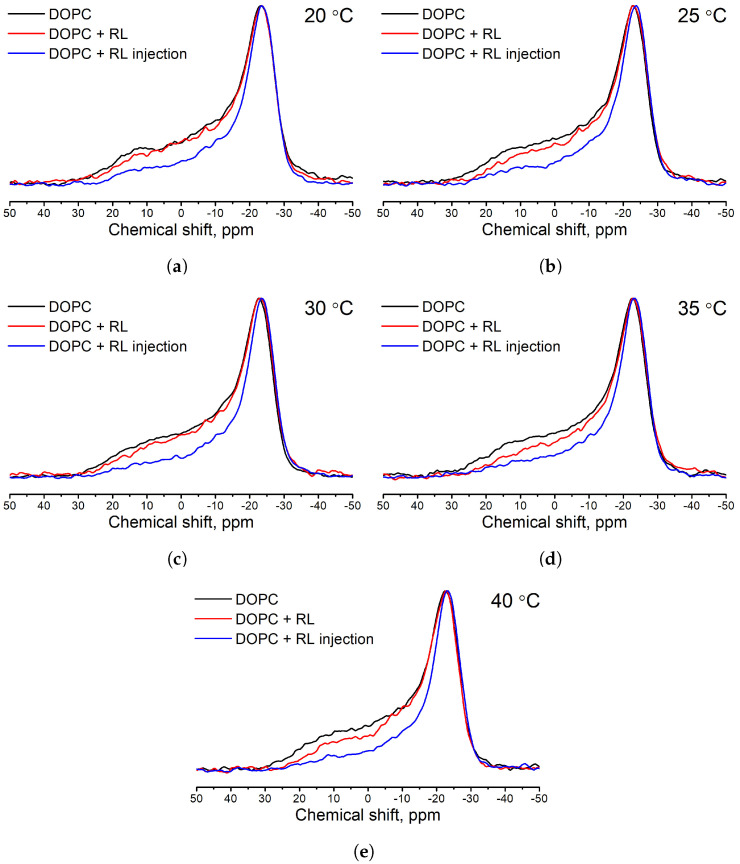
${}^{31}$P NMR spectra of liposomes from DOPC (black solid line), liposomes from DOPC and rhamnolipids (red solid line), and liposomes from DOPC with rhamnolipid injection (blue solid line) at different temperatures (**a**–**e**).

In the first part of the work, a study of the interaction of a rhamnolipids mixture with DOPC membranes depending on the method of introducing rhamnolipids was made. Samples of liposomes from pure DOPC and two samples of liposomes from DOPC with the addition of rhamnolipids in various methods were studied. Figure 2 shows the ${}^{31}$P NMR spectra of the studied samples at various temperatures. For all samples, an anisotropic powder-like spectrum is observed without a pronounced isotropic line. The spectrum can be described by a diagonal chemical shift tensor with parameters $\sigma_{xx}=\sigma_{⊥}$, $\sigma_{yy}=\sigma_{⊥}$, and $\sigma_{zz}=\sigma_{\|}$ (asymmetry parameter η=0). This form of the spectrum is typical for the case of liposomes and other aggregates of lipid membranes in the liquid crystal phase [43,46], the absence of an isotropic line in the spectrum indicates that all phospholipid molecules are in the bilayer structure.

A qualitative comparison of the spectra of samples prepared by different methods suggests that the spectrum of liposomes prepared from a mixture of DOPC and RL practically coincides with the spectrum of a control sample prepared only from DOPC, especially at temperatures close to room temperature. On the other hand, it is clearly seen that the spectrum of the sample prepared by RL injection differs significantly from the spectrum of the control sample in the region of the anisotropic shoulder—the intensity of the shoulder for DOPC liposomes with RL injection is noticeably lower than the control spectrum shoulder. A decrease in the intensity of the anisotropic shoulder of the spectrum with the addition of rhamnolipids may indicate either an increase in the size of liposomes [41,47] or a direct change in the curvature of the liposome upon the incorporation of rhamnolipids into the membrane due to steric interactions [48].

**Figure 3:**
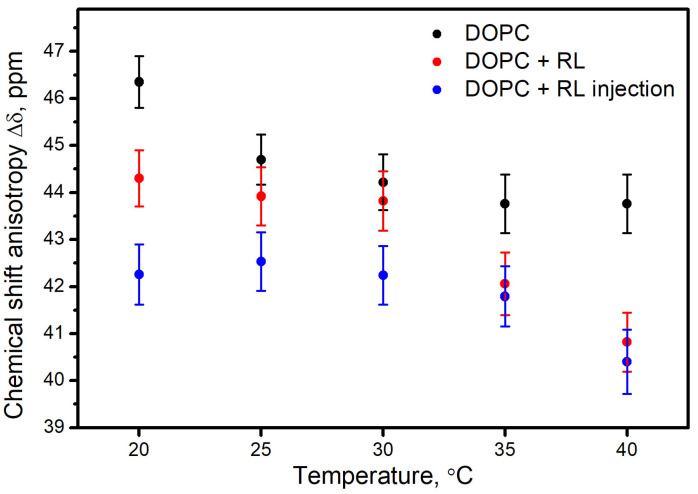
Temperature dependence of chemical shift anisotropy parameter Δδ for liposomes from DOPC (•), for liposomes prepared from a mixture of DOPC and rhamnolipids (•) and for liposomes from DOPC with rhamnolipids injection (•).

For the purpose of quantitative comparison, each experimental spectrum was approximated in the DMfit software and the chemical shift anisotropy Δδ was determined (the results are shown in Figure 3). Regardless of the method of preparation, the presence of rhamnolipids contributes to a decrease in CSA Δδ compared to the spectrum of the control sample. A decrease in the values of CSA Δδ with the presence of rhamnolipids means an increase in the rotational mobility of the head group of phospholipids [36,49,50,51,52]. However, the magnitude of this effect depends on the way rhamnolipids are added. The Δδ values for liposomes prepared from a mixture of DOPC and rhamnolipids (red dots) turned out to be on average less than the Δδ values for the control sample (black dots) by 1.57 ppm, and for DOPC liposomes with an aqueous injection of rhamnolipids (blue dots) they were less at 2.71 ppm.

An increase in the rotational mobility of the phospholipid head group can be explained by an increase in the average distance between phospholipid molecules upon the incorporation of rhamnolipid molecules into the membrane. At the same time, in samples prepared from a mixture of DOPC + RL, rhamnolipid molecules are probably located deeper in the bilayer of the membrane and therefore have less effect on the mobility of the head group.

**Figure 4:**
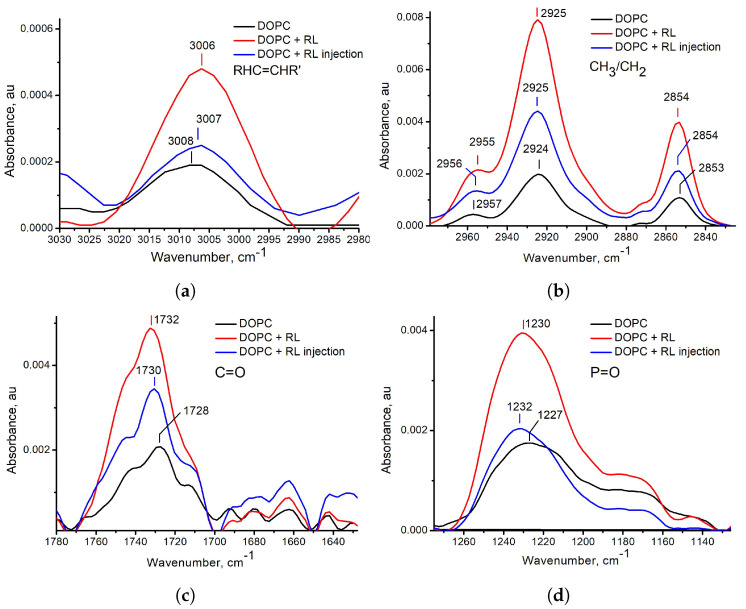
ATR−FTIR spectra of liposomes from DOPC without rhamnolipids (black solid line), liposomes prepared from a mixture of DOPC and rhamnolipids (red solid line), and liposomes from DOPC with an rhamnolipids injection (blue solid line) in the region of hydrogen vibrations at a double carbon bond in the cis configuration (**a**), vibrations in CH${}_{2}$ and CH${}_{3}$ groups (**b**), vibrations of C=O (**c**) and P=O (**d**) bonds.

The ATR-FTIR spectra of the above three samples of DOPC and RL liposomes are presented in Supplementary materials in Figure S2. A number of sources were used to decipher the spectrum [36,49,53,54,55]. Only some vibrations that characterize interactions in the hydrophobic (hydrogen vibrations in CH${}_{3}$, CH${}_{2}$ groups and at the double bond in the acyl chain) and hydrophilic parts of the membrane (C=O and P=O double bond vibrations) are sensitive to the incorporation of exogenous molecules into the membrane [36,56]. Fragments of FTIR spectra in the range of wavenumbers of vibrations of such groups with marked values of absorption peaks are shown in Figure 4.

Let us consider the shifts in the characteristic vibration frequencies, which reflect changes in the hydrophobic part of the membrane. For hydrogen vibrations in the double bond HRC=CR’H and in the CH${}_{3}$ group, the presence of RL results in a shift in the vibration frequency to the lower wavenumbers (Figure 4a,b), which means an increase in order in the region of these bonds. The shift for vibration at the double bond is explained by the direct incorporation of rhamnolipids into the membrane: the double bond in DOPC is located between the 9th and 10th carbons, while the length of the acyl chain of the rhamnolipid used in this work is 10 hydrocarbon units. As for the shift of the vibration of the CH${}_{3}$ group, the possibility of an increase in order in the middle of the membrane, where this group is located, is noted in the literature [2]. At the same time, the upward shift in wavenumbers for the CH${}_{2}$ group (Figure 4b) indicates that, in general, the acyl chain has become more conformationally disordered [29,46,57].

For vibrations that characterize the hydrophilic part of the membrane with the addition of rhamnolipids, a shift in vibration frequencies higher wavenumbers is observed (Figure 4c,d). Such a shift is explained by the strong polarity of the rhamnose regions of rhamnolipids, which form a large number of hydrogen bonds with the surrounding water, pulling water molecules away from the carboxyl and phosphate groups of the phospholipid. A decrease in the degree of lipid hydration leads to an increase in the vibration frequency for C=O and P=O bonds [36,55,58].

Note that downward shifts of the frequencies of HRC=CR’H and CH${}_{3}$ vibrations turned out to be higher for the sample of liposomes prepared from a mixture of DOPC and rhamnolipids, and the shift to the region of large wave numbers for vibrations of the C=O bond turned out to be higher. And for liposomes from DOPC with an aqueous injection of rhamnolipids, the shift for vibrations of the P=O bond turned out to be higher. Since the HRC=CR’H, CH${}_{3}$, and C=O groups are located deeper in the membrane than the P=O group, then for liposomes prepared from a mixture of DOPC and rhamnolipids, we can say that rhamnolipids in these samples are located deeper in the membrane than for sample with rhamnolipids injected.

**Figure 5:**
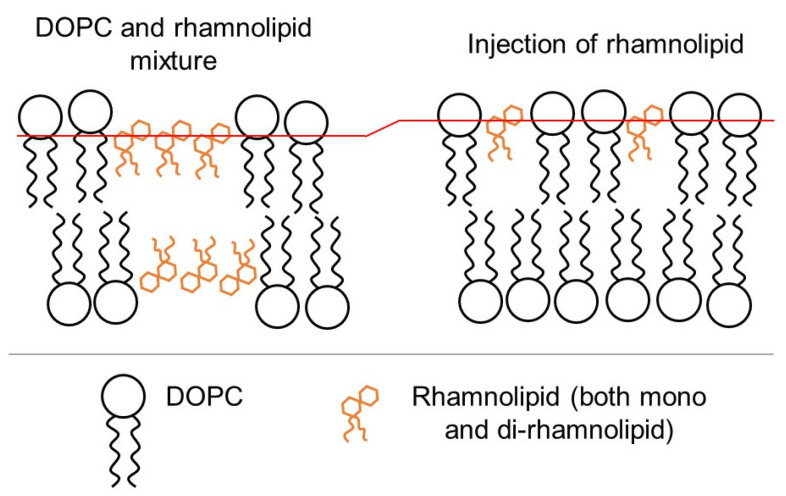
A possible model for the incorporation of rhamnolipids with a model DOPC membrane depending on the preparation protocol.

The data demonstrate changes in the NMR and FTIR spectra from chemical groups along the entire length of the phospholipid molecule, therefore, it can be concluded that, regardless of the preparation protocol, rhamnolipids are incorporated into the model membrane. The tendency of rhamnolipid molecules to incorporate into membranes is also described in the literature [12]. The difference in the observed data for samples prepared according to different protocols can be explained by the following considerations illustrated on Figure 5.

It should be assumed that stable rhamnolipid clusters can form from a lipid mixture during the preparation of liposomes by the film method. Such clusters can form at the stage of dissolution of the initial mixture or evaporation of the solvent (chloroform and methanol) and the formation of a film on the flask wall. Taking into account that rhamnolipids are surfactants and can form micelles, this is a very likely assumption. Similar clusters were studied by Oliva et al [33]. Subsequently, these rhamnolipid clusters could be incorporated into model membranes at the stage of lyophilization or at the stage of closing film fragments into liposomes in water. Such clusters should be located in all lamellae of liposomes, and, according to FTIR data and taking into account the smaller size of rhamnolipid molecules, they should occupy a deeper position in the membrane. In the case of liposomes with rhamnolipids prepared according to the injection protocol, the injection solution should also contains stable rhamnolipid aggregates, from which rhamnolipid molecules could randomly migrate [28] as monomers and integrate the outer lamellae of the DOPC liposome closer to the head of the phospholipids. Proposed types of rhamnolipids incorporation into the model membrane explains/does not contradict the data observed and the literature data.

### 3.2. Influence of the Presence of Sterols

**Figure 6:**
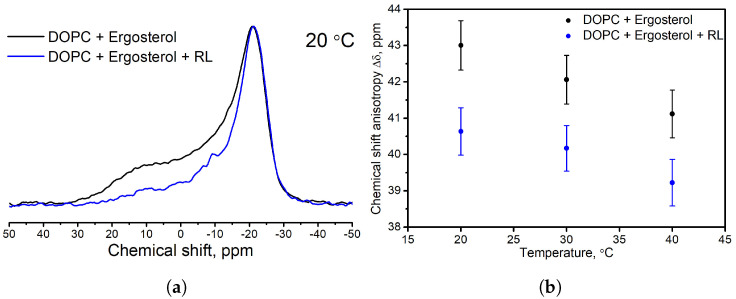
${}^{31}$P NMR spectrum of liposomes from DOPC with the addition of ergosterol without rhamnolipids (black solid libe) and with an aqueous injection of rhamnolipids (blue solid line) at a temperature of 20 ${}^{\circ }$C (**a**). Dependence of CSA values Δδ on temperature (**b**).

In the second part of the work, a study of the interaction of a mixture of rhamnolipids with DOPC membranes with different sterol compositions was made. Samples of liposomes from DOPC and one of the sterols were studied: ergosterol, cholesterol, lanosterol. One part of these samples was left as control samples, and an aqueous solution of a mixture of rhamnolipids was introduced into the other. Figure 6a shows ${}^{31}$P NMR spectra of DOPC liposome samples with ergosterol before and after RL injection. With the addition of RL, the effect that we saw above is observed: the intensity decreases in the region of the anisotropic shoulder, and the anisotropy of the chemical shift Δδ decreases (on average by 2.05 ppm) over the entire temperature range studied (Figure 6b). Therefore, we can again draw conclusions about an increase in the size of liposomes and an increase in mobility in the head group of the phospholipid in the presence of RL.

**Figure 7:**
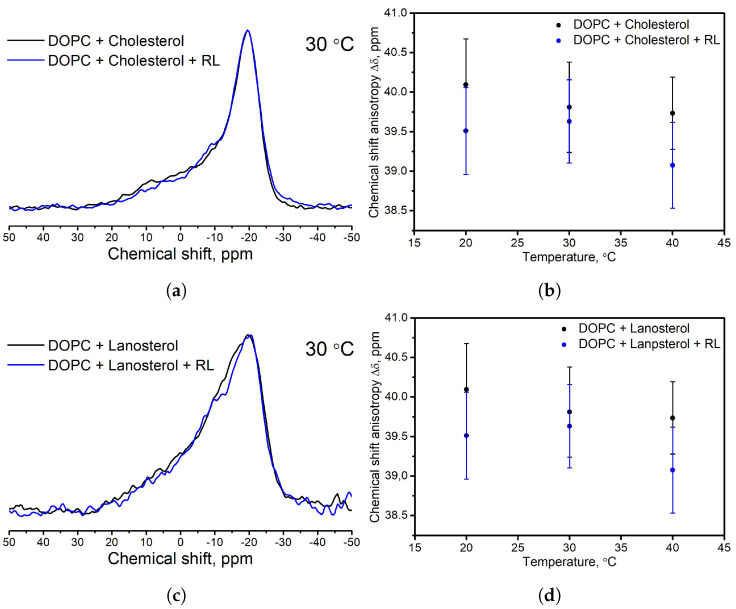
${}^{31}$P NMR spectra of liposomes based on DOPC (black solid line), liposomes based on DOPC and injected with rhamnolipids (blue solid line) containing cholesterol (**a**) and lanosterol (**c**). Dependences of CSA Δδ values on temperature for samples with cholesterol (**b**) and lanosterol (**d**).

At the same time, the NMR spectra of samples with cholesterol and lanosterol practically did not change after the injection of RL. The ${}^{31}$P NMR spectra of these samples at a temperature of 30 ${}^{\circ }$C and the temperature dependences of CSA Δδ values are shown in Figure 7. Therefore, we can say that there is no effect of RL on the size of liposomes and mobility in the head group of the phospholipid in the presence of cholesterol and lanosterol in the membrane.

**Figure 8:**
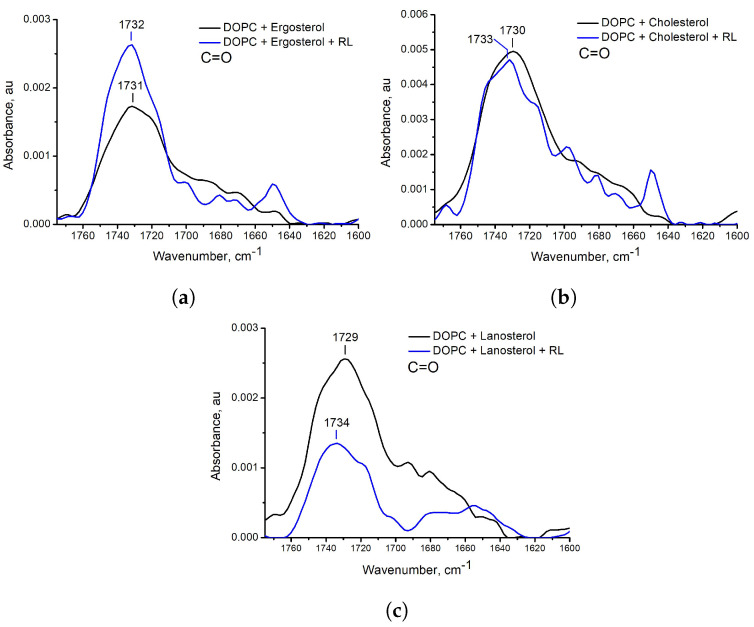
ATR−FTIR spectra in the region of C=O vibration for DOPC liposomes with ergosterol (**a**), cholesterol (**b**), and lanosterol (**c**) without rhamnolipids (black solid line) and with aqueous injection of rhamnolipids (blue solid line).

**Figure 9:**
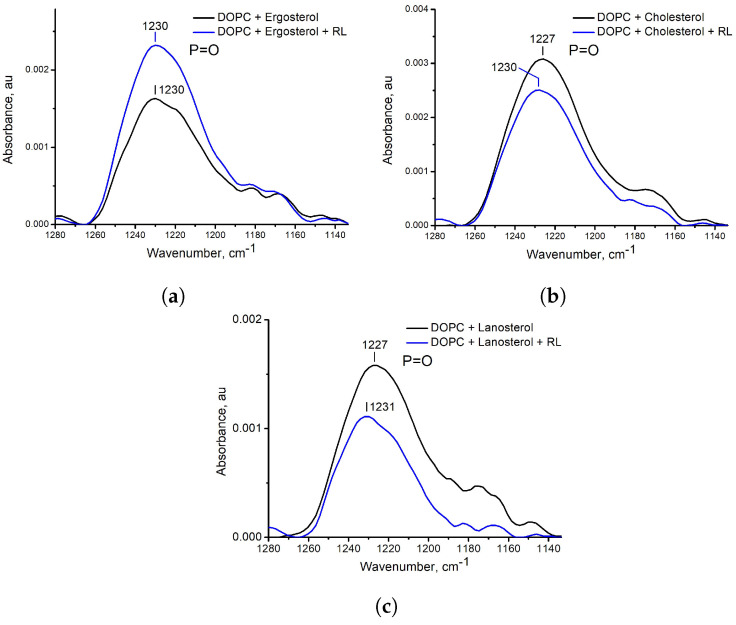
ATR−FTIR spectra in the region of P=O vibration for DOPC liposomes with ergosterol (**a**), cholesterol (**b**), and lanosterol (**c**) without rhamnolipids (black solid line) and with aqueous injection of rhamnolipids (blue solid line).

The results of FTIR spectroscopy turned out to be opposite (data for polar vibrations are shown in Figure 8 and Figure 9, as well as full spectra in Supplementary materials, Figures S3 and S4). In the samples with ergosterol in the presence of RL, the shift in the frequencies of vibrations of the C=O bond was 1 cm${}^{-1}$ upwards and did not change at all for the P=O bond and CH${}_{2}$, CH${}_{3}$ bonds. However, for samples with cholesterol and lanosterol in the presence of RL, an upward shift of vibration frequencies is observed: for C=O bond 3 and 5 cm${}^{-1}$, respectively, for P=O bond vibrations 3 and 4 cm${}^{-1}$, respectively. The asymmetric stretching vibrations of the CH${}_{2}$ group for samples with cholesterol and lanosterol after the addition of RL shifted by 1 cm${}^{-1}$ to the region of higher values and by 1 cm${}^{-1}$ to the region of lower values for the asymmetric vibration of the CH${}_{3}$ group.

**Figure 10:**
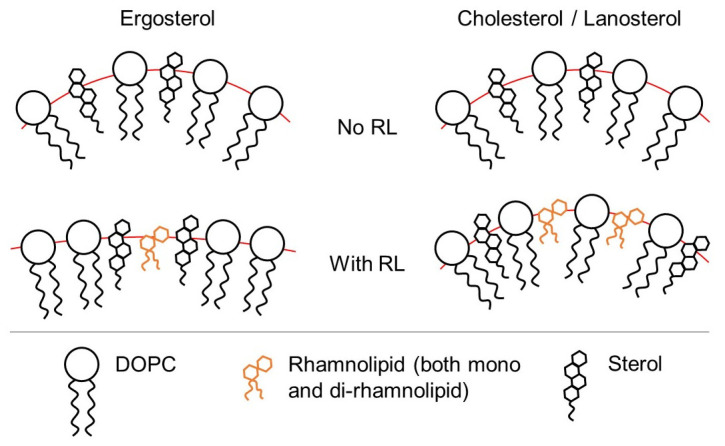
A possible model for the incorporation of rhamnolipid molecules with a model DOPC membrane depending on the sterol composition of the membrane.

Summarizing, in samples of liposomes with ergosterol, according to NMR, an increase in the rotational mobility of the head group of phospholipids and changes in the curvature of liposomes as a whole are observed, and according to FTIR, there is no effect of rhamnolipids on groups of phospholipid molecules. This can be explained by the presence of a special interaction between rhamnolipids and ergosterol, which apparently leads to the fact that rhamnolipid molecules are inserted near ergosterol, thereby ergostreol shields membrane phospholipids from the action of rhamnolipids. On the other hand, for samples of liposomes with cholesterol and lanosterol, no changes are observed in the NMR spectra, however, the FTIR spectra demonstrate the effect of rhamnolipids on the vibrations of phosphogroup and the DOPC core. The same situation was observed when rhamnolipids were added to pure DOPC membranes. In this case—different sterol composition of the model membranes—one must assume the following types (Figure 10) of the interaction of rhamnolipids with DOPC model membranes, which does not contradict the data observed.

However, it should be remembered that phospholipid-sterol systems are quite complex. The effects in such systems may depend on various parameters, ranging from the length of the hydrocarbon chain of the phospholipid to the properties of the membrane as a whole [37].

## 4. Conclusions

In this work effects of natural rhamnolipid mixture produced by bacteria of the rhizosphere zone of plants *Pseudomonas aeruginosa* on dioleoylphosphatidylcholine model membrane depending on method of preparation and sterol content were studied. It was shown that, depending on the method of adding rhamnolipids to the model membrane, rhamnolipid molecules are incorporated into the membrane structure in different ways. In the case of preparation from a mixture, rhamnolipids are probably incorporated into the membrane in the form of clusters and are located closer to the middle of the bilayer; in the case of the preparation by injection, rhamnolipid molecules migrate into the membrane in the form of separate molecules and are located closer to the head part of phospholipids. This type of incorporation of all others considered in our opinion does not contradict observed and the literature data. From the point of view of practical application, the only way of using rhamnolipids in biotechnology is the injection of rhamnolipids, and, since the way in which rhamnolipids are added to the membrane is important, the biological effects caused by rhamnolipids should be further investigated in systems prepared by injection.

The sterol composition of the model membrane also affects the interaction of rhamnolipids with the membrane. In the case of model membranes with ergosterol, rhamnolipid molecules are likely to be embedded in the regions where ergosterol is located, which suggests the presence of a specific interaction between rhamnolipids and ergosterol. In the case of model membranes with cholesterol and lanosterol, rhamnolipid molecules are likely to be integrated into the regions of phospholipids, which, however, does not lead to a change in the size of the liposomes and the mobility of the head group of phospholipids. The significance of this result lies in the fact that some cells whose membranes have a certain sterol composition can be protected from the influence of rhamnolipids, and vice versa, the membranes of other cells can be weakened by the introduction of rhamnolipids and may become less effective against drugs and viruses, which could be useful for biocontrol in agriculture. On the other hand, a controlled addition of rhamnolipids and controlled sterols composition could be used to create liposomes with desired properties for drug delivery.

## Data Availability

Data is contained within the article or supplementary material.

## Supplementary Materials

The following supporting information can be downloaded at: https://www.mdpi.com/article/10.3390/membranes13010112/s1, Figure S1: Example of approximation of ${}^{31}$P-NMR spectra of liposomes from DOPC and rhamnolipids, liposomes from DOPC with aqueous injection of rhamnolipids; Figure S2: ATR-FTIR spectra of liposomes from DOPC without rhamnolipids, liposomes from DOPC and rhamnolipids, and liposomes from DOPC with injection of rhamnolipids; Figure S3: ATR-FTIR spectra for DOPC liposomes with ergostero, cholesterol, and lanosterol without the addition of rhamnolipids; Figure S4: ATR-FTIR spectra for DOPC liposomes with ergostero, cholesterol, and lanosterol with aqueous injection of rhamnolipids.

## Abbreviations

The following abbreviations are used in this manuscript:

DOPC	Dioleoylphosphatidylcholine
RL	Rhamnolipid
NMR	Nuclear magnetic resonance
ATR-FTIR	Attenuated total reflection Fourier transform infrared spectroscopy
