# Supplementary material for: Effects of Natural Rhamnolipid Mixture on Dioleoylphosphatidylcholine Model Membrane Depending on Method of Preparation and Sterol Content
Source: Membranes (Basel). 2023 Jan 15;13(1):112. doi: 10.3390/membranes13010112 (PMC9865241; doi:10.3390/membranes13010112)
Supplement: Supplementary file 1 [file membranes-13-00112-s001.zip › membranes-2082940-supplementary.pdf]

membranes

Supplementary Materials

Effects of Natural Rhamnolipid Mixture on Dioleoylphosphatidylcholine Model Membrane Depending on Method of Preparation and Sterol Content

Konstantin Potapov [1], Alexander Gordeev [2], Liliya Biktasheva [2], Maya Rudakova [3] and Artem Alexandrov [1,*]

[1] Department of Molecular Physics, Institute of Physics, Kazan Federal University, 420011 Kazan, Russia
[2] Institute of Environmental Sciences, Kazan Federal University, 420011 Kazan, Russia
[3] Institute of Information Technology and Intelligent Systems, Kazan Federal University, 420011 Kazan, Russia
* Correspondence: alexandrov.artem.sergeyevich@gmail.com

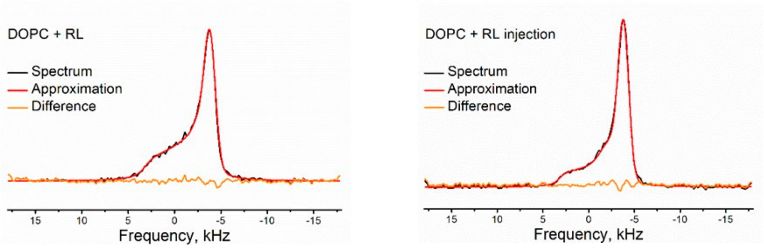

Figure S1. Example of approximation of ^{31}P-NMR spectra of liposomes from DOPC and rhamnolipids, liposomes from DOPC with aqueous injection of rhamnolipids.

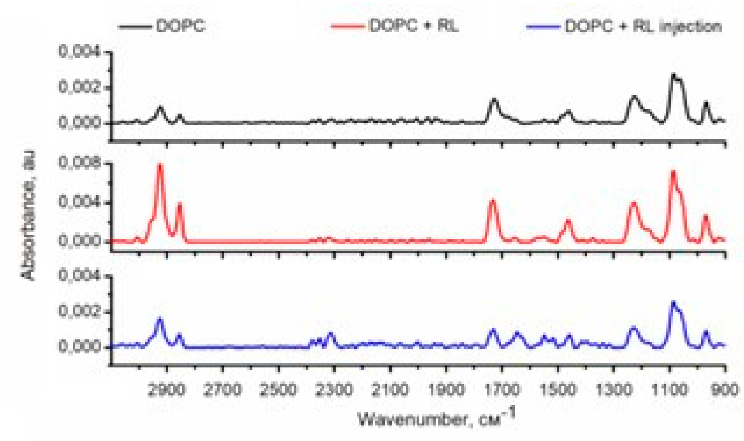

Figure S2. ATR-FTIR spectra of liposomes from DOPC without rhamnolipids, liposomes from DOPC and rhamnolipids, and liposomes from DOPC with injection of rhamnolipids.

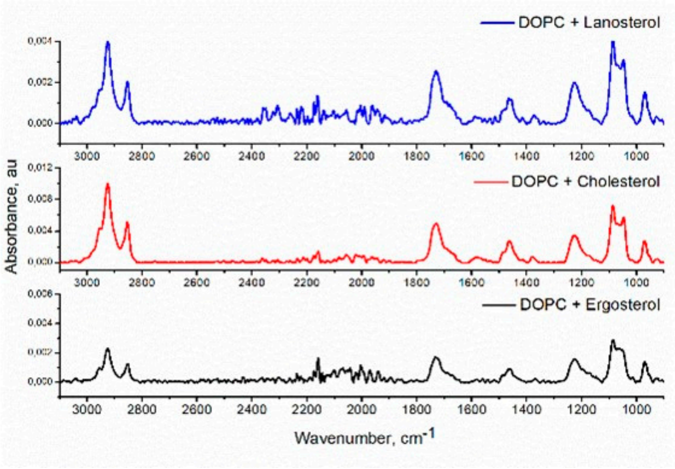

Figure S3. ATR-FTIR spectra for DOPC liposomes with ergostero, cholesterol, and lanosterol without the addition of rhamnolipids.

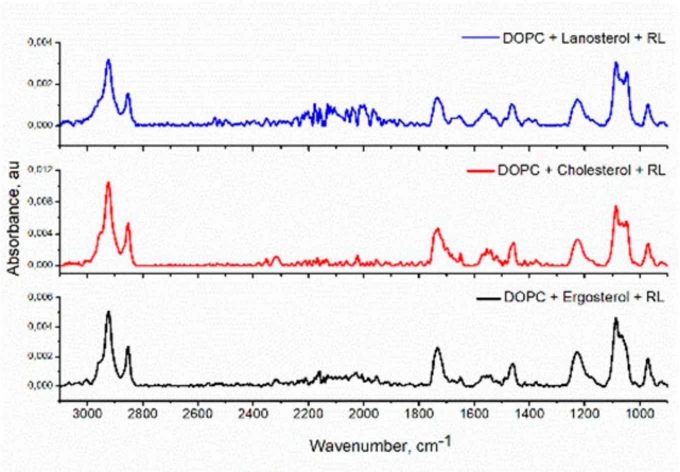

Figure S4. ATR-FTIR spectra for DOPC liposomes with ergostero, cholesterol, and lanosterol with aqueous injection of rhamnolipids.
